# Lung adenocarcinoma in the era of targeted therapies: histological classification, sample prioritization, and predictive biomarkers

**DOI:** 10.1007/s12094-012-0983-z

**Published:** 2013-01-29

**Authors:** E. Conde, B. Angulo, E. Izquierdo, L. Paz-Ares, C. Belda-Iniesta, M. Hidalgo, F. López-Ríos

**Affiliations:** 1 Laboratorio de Dianas Terapéuticas, Centro Integral Oncológico Clara Campal, Hospital Universitario Madrid Sanchinarro, Faculty of Medicine, Universidad San Pablo-CEU, Madrid, Spain; 2 Department of Oncology, Instituto de Biomedicina de Sevilla (IBIS) and Hospital Universitario Virgen del Rocío, Sevilla, Spain; 3 Department of Oncology, Centro Integral Oncológico Clara Campal, Hospital Universitario Madrid Sanchinarro, Faculty of Medicine, Universidad San Pablo-CEU, Madrid, Spain

**Keywords:** Lung cancer, Biomarkers, EGFR, ALK

## Abstract

The arrival of targeted therapies has presented both a conceptual and a practical challenge in the treatment of patients with advanced non-small cell lung carcinomas (NSCLCs). The relationship of these treatments with specific histologies and predictive biomarkers has made the handling of biopsies the key factor for success. In this study, we highlight the balance between precise histological diagnosis and the practice of conducting multiple predictive assays simultaneously. This can only be achieved where there is a commitment to multidisciplinary working by the tumor board to ensure that a sensible protocol is applied. This proposal for prioritizing samples includes both recent technological advances and the some of the latest discoveries in the molecular classification of NSCLCs.

## Introduction

The classification of primary lung carcinomas is probably one of the least precise among all the solid tumors. Paradoxically, however, it is one of the most reproducible. The lung pathology field has not only been static, as recently mentioned, but also perceived as uninteresting [1]. This has come about due to a set of factors which can be better understood from a historical perspective. The discovery that some of these carcinomas, i.e., small cell carcinomas (SCLCs), responded incredibly well to chemotherapy led to a lack of interest in the correct classification of lung carcinomas. It is a situation that continues even today. This therapeutic discovery gave rise to the use of the intelligent term “non-small cell lung carcinoma (NSCLC)” and to a tolerance of the lack of precision. According to Edwards et al., this nomenclature was suggested in 1984 by Chuang et al. and accepted from then on during the pre-immunohistochemistry (IHC) era to decrease the risk of over-interpreting small biopsy specimens [2–4]. Over the years, the term has been extended to surgical specimens, clinical trials, etc. [5, 6].

The term “large cell carcinoma (LCC)” was defined by the 2004 WHO classification as one of exclusion [7]. It is also misused for two main reasons:

- In the setting of surgical specimens, it gives rise to the possibility of giving such a name to all difficult cases with no obvious keratin (squamous cell carcinomas, SCCs) or gland formation (adenocarcinomas, ACs).
- It is frequently applied as a synonym of non-small cell carcinoma in small thoracic biopsies [7–9].

A few years ago, there was a sudden interest, which has continued to grow, in the histology of lung cancer [10]. This can be explained by various factors. Firstly, ACs have treatable molecular alterations: mainly *EGFR* mutations and *ALK* translocation [11–14]. Secondly, some targeted agents should not be used in SCCs. This is not only because they do not provide better response rates (pemetrexed), but also because their use in this histological type is associated with life-threatening complications (i.e., bevacizumab) [15–17]. As if we had not learned our lesson previously, another imprecise term is emerging: “non-squamous histology” [15, 18].

As such, the current therapeutic situation of lung cancer demonstrates that establishing a treatment requires a precise histological diagnosis. Many treatable molecular alterations are linked to specific histological types. Equally, diagnosis must be reasonable. If we exhaust the sample during classification, we will not be able to produce predictive biomarkers [19, 20]. Certain considerations are, therefore, necessary. Although there appears to be complete specificity for AC or SCC for several driver mutations, some poorly differentiated ACs may be classified as SCC due to the presence of squamoid features [21]. Moreover, a significant number of those poorly differentiated ACs may have treatable molecular alterations [22]. For example, up to 18 % of so-called squamoid subtype ACs can harbor *EGFR* mutations [23]. The practical implication is that it is probably better to use the “NSCLC-NOS” (not otherwise specified) category when there is inconclusive evidence of squamous or glandular differentiation.

## Lung carcinoma classification for non-pathologists

In the paragraphs that follow, we shall discuss certain aspects which are, perhaps, less well-known to clinicians or pathologists with less experience in thoracic pathology.

While SCLCs are not the subject of this study, it is useful to include a paragraph about them. Currently, we recommend confirmation with IHC of all SCLCs which appear as such in the study with hematoxylin and eosin (H&E), i.e., scant cytoplasm, granular nuclear chromatin with nucleoli inconspicuous or absent and high mitotic rate. For the IHC confirmation, the following are sufficient: two neuroendocrine differentiation markers (CD56, chromogranin or synaptophysin); a pankeratin AE1–AE3 with frequent “dot-like” pattern; TTF-1 (70–90 % positive) and a very high proliferative index by Ki-67 (70–100 %). This is very important in making the differential diagnosis with lymphoma, melanoma, and other lung neoplasias: poorly differentiated SCCs, large neuroendocrine cell carcinoma, and carcinoid tumors, especially [24, 25]. Two aspects must be taken into account:

- Neoplasias with neuroendocrine differentiation can show positivity for p63. This includes some 77 % of SCLCs [26].
- SCLCs observed outwit the typical bronchial biopsy with crush artifact, and chromatin stretching have an appearance with which we, pathologists, are not familiar, i.e., very well-conserved cells with visible nucleus, greater size, etc. Examples might include SCLCs observed in a pulmonary core-needle biopsy, in a wedge lung resection specimen or in a metastasis. These unfamiliar appearances can present huge difficulties in differential diagnosis [25].

Furthermore, ACs and SCCs represent the two major types of NSCLCs. Distinguishing between the two histological types can appear extremely difficult by routine light microscopy, particularly in small biopsies and cytology samples. This can affect up to 35 % of cases [27]. In the case of NSCLCs, the use of IHC is essential where a specific, conclusive diagnosis cannot be produced. Indeed, we can only make such a diagnosis where we identify keratinization in SCCs or gland formation in ACs. As such, probably the most accepted antibody pair in the literature is that formed by TTF-1 (marker of glandular differentiation) and p63 (marker of squamous differentiation) [28]. In additional, SCCs also tend to be positive for desmocollin-3 (the most specific marker) and CK5/6 (the most sensitive marker) [27]. ACs, however, stain with Napsin A (the most specific marker) and CK7 (the most sensitive marker) [27]. Interestingly, desmocollin-3 (*DCS3*) was the top differentially expressed gene in our own microarray comparison between lung ACs and SCCs [29]. This finding has been independently confirmed by other groups [27, 30].

It must be stressed that analytical and post-analytical aspects (interpretation) can influence the supposed specificity and sensitivity of an antibody. Given this consideration, it is useful, for example, to recall that genuine staining of p63 must be intensive and extensive. Faint or focal immunostaining for p63 should be considered non-specific until there is proof that it is not [9, 27, 28, 31–35]. Recent studies show the value of p40 (∆Np63) as a more specific marker of squamous differentiation [36–38]. Another idea is to use double staining protocols combining a nuclear and a cytoplasmic/membranous antibody (for example, CK7-p63 and CK5/6-TTF-1) to reduce the number of slides necessary [27]. However, we should always bear in mind that methodologies taken to raise specificity may also lower the likelihood of clinical application. For example, new antibodies or protocols may not be easy to implement or interpret.

## Sample prioritization

**Fig. 1:**
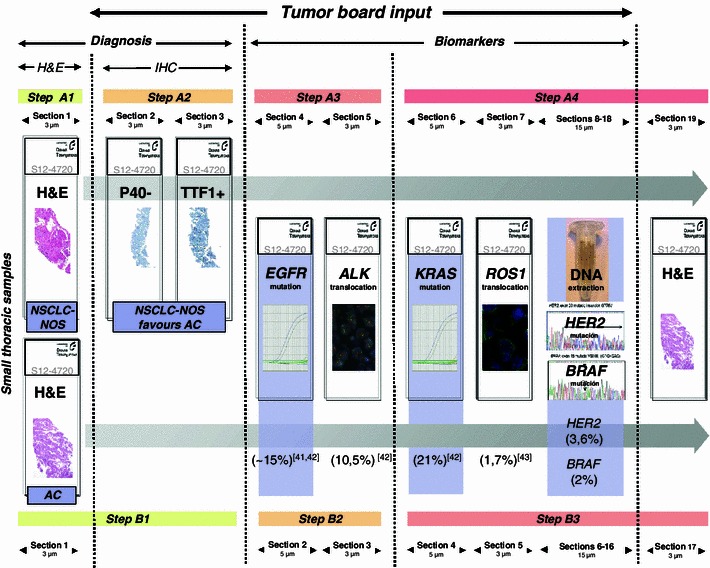
A realistic approach for sample prioritization for the study of predictive biomarkers in patients with advanced lung ACs. Route A is for cases that require classificatory IHC while route B is for cases that are diagnosed based on the H&E alone. The relative frequency of the different genetic alterations is shown in parenthesis. Data from *ROS1* translocation is taken from the literature [43]. The other percentages come from our own experience [41, 42], and unpublished data

While we firmly believe as pathologists in the importance of the correct histological subtyping of carcinomas of the lung, we also understand that it is not always possible in the real world. There is currently no consensus on how to prioritize the different predictive assays that are frequently performed in different laboratories using different approaches. These include, for example, H&E and IHC for diagnosis, accurate histological subtyping and some predictive biomarkers, fluorescence in situ hybridization (FISH) for *ALK* translocation, and PCR for *EGFR* mutations [35, 39, 40]. The “tissue sparing” algorithm that we propose for molecular tests in small thoracic samples is depicted in Fig. 1 [41–43]. This approach not only saves time and tissue, but will also provide realistic information on the true incidence and overlap of the different molecular alterations. These considerations are not merely academic. Rather, they influence the cost of drugs and their biomarkers [44].

In order to minimize the loss of material, the following are suggested:

1. Sufficient multidisciplinary communication, oncological, and pathological to put the paraffin block in the microtome as few times as possible;
2. Reasonable use of classificatory IHC with a restricted panel of antibodies. While this increases the percentage of “NSCLC-NOS”, the sample is preserved for future studies on therapeutic targets [27, 35].

**Fig. 2:**
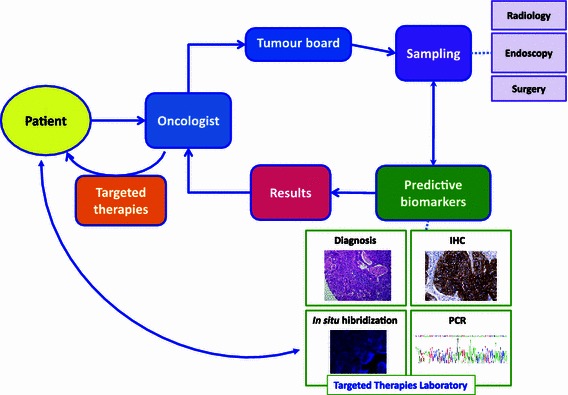
A simplified view of an integrated pathway for the study of predictive biomarkers in patients with advanced NSCLCs. There are two interesting aspects. **a** If the sampling is managed by the tumor board, there will be better sample prioritization than has happened previously. **b** There is an increased awareness of technological advantages and disadvantages among patients and their families

Figure 2 shows how sample prioritization and biomarker information are integrated into patient care.

## Predictive biomarkers

As a result of our experience in recent years, we believe that a protocol similar to that depicted in Fig. 1 would provide information on multiple predictive biomarkers [22, 28, 41, 42, 45]. Although some aspects of our approach have already been implemented, others remain theoretical [42]. Ideally, the protocol should be validated prospectively with an intention-to-treat philosophy. Our protocol has several advantages. Firstly, the sample is sectioned in as few steps as possible to decrease tissue waste at the microtome and also to shorten the turn-around time. Secondly, this approach permits the combination of different methodologies: IHC, in situ hybridization and PCR. This is important because, currently, it is not realistic to “multiplex” the analytical phase of, for example, mutation, amplification, translocation, and overexpression detection. Thirdly, it allows the order of the different biomarkers to be changed depending on estimated prevalence, histological characteristics, drug approvals or access to clinical trials, or compassionate use. For example, ACs with lepidic (formerly “bronchioloalveolar”), papillary or micropapillary components are commonly associated with *EGFR* mutations, papillary growth has also been linked with *BRAF* V600E mutated ACs, and signet-ring cells are typically present in *ALK* positive lung ACs [46–49]. Fourthly, it also allows the methodology to be changed. This is feasible because most IHC and in situ hybridization tests are performed on 3–5 μm sections, and there are recent PCR methods that allow mutation testing from a single 5 μm section [50–52]. Fifthly, simultaneous testing of at least some of the biomarkers (those included in step A3/B2 and step A4/B3) will give us the response as to whether they are truly mutually exclusive or not, as well as the true prevalence in a given population. If they are not mutually exclusive, we will not leave potentially positive patients without testing. This is especially important now as, where methodological caveats can be reasonably discounted, we are beginning to accept the existence of:

- Molecular heterogeneity: recently published discordance rates between primary tumors and metastases were 14 % for *EGFR* mutations and 13.5 % for *ALK* overexpression [53, 54];
- Overlap in predictive markers. For example, *ALK* translocations may be identified in both *EGFR* and *HER2* mutant patients [55, 56].

Ideally, an H&E is conducted first to confirm the malignant nature of the disease. If the tumor shows malignant glands, an AC can be diagnosed with certainty. If not, a couple of classificatory IHC stains is probably sensible (step A2). Step A3/B2 involves simultaneous testing for *EGFR* mutations and for *ALK* translocation. Almost everyone will agree that, in patients with advanced lung ACs, it is necessary to know those two biomarkers. However, there is no consensus on whether testing should be simultaneous or sequential. Nor is there consensus on the methodology to be used [35, 39, 57, 58]. A wide variety of methods have been applied to *EGFR* mutation analysis. Although direct sequencing is still probably the most frequently used method, in recent years commercial real time PCR assays have become increasingly popular [41, 45]. Although FISH is currently the only approved method to identify potential responders to crizotinib through the presence of the *ALK* translocation, ALK IHC may also prove useful in this setting [57].

The remaining biomarkers (step A4/B3) represent a more innovative approach to lung cancer targeted therapies. It is probably wise to check the status of *KRAS* [59]. In addition, patients with *ROS* rearrangements may also respond to crizotinib [43]. We should also extract DNA again and look for mutations in *HER2* and *BRAF* [60–62]. A final H&E section will re-assure us as to how many tumor cells we have left for the latter assays and the possibility of producing more biomarkers in the future (i.e., *MET* amplification, MET overexpression, and *RET* rearrangement) [63–65].

To conclude, the final consideration is: what is the minimum number of tumor cells needed to conduct these tests? For IHC, counts of 2,000 tumor cells are recommended [66]. In the case of FISH, 100 cells are sufficient [50, 66]. For PCR techniques, it is very important to know their limit of detection (LOD) and to work with a safety margin. For example, if the LOD of direct sequencing is 30 %, the percentage of tumor cells should ideally be 40–50 % [41, 45, 66, 67]. In the case of real time PCR with a LOD of 1–5 %, we must be aware of the risk of false negatives when the percentage of tumor cells is below 10 % [41, 45, 66, 67].

Unfortunately, most of the advances in personalized treatment of NSCLCs have been confined to the treatment of patients with ACs. However, better molecular characterization of SCCs is enabling this subgroup to become a growing area of interest. As such, specific molecular defects such as *FGFR1* amplifications, *DDR2* mutations, *PI3KCA* amplifications and mutations will become part of the routine molecular diagnostic workup of SCCs [68–71]. Lessons learned from ACs should help to make these exciting findings a success story for the treatment of SCCs.

In conclusion, we have presented a realistic approach to lung AC targeted therapies. This includes accurate histological subtyping, as well as sample prioritization to release information on as many predictive biomarkers as possible.
